# Use of Implantable Cardioverter-Defibrillators for Primary Prevention of Sudden Cardiac Death in Asia

**DOI:** 10.1016/j.jacasi.2023.02.004

**Published:** 2023-04-25

**Authors:** Nobuhiko Ueda, Takashi Noda, Kengo Kusano, Satoshi Yasuda, Takashi Kurita, Wataru Shimizu

**Affiliations:** a Department of Cardiovascular Medicine, National Cerebral and Cardiovascular Center, Suita, Japan; b Department of Cardiovascular Medicine, Tohoku University Graduate School of Medicine, Sendai, Japan; c Department of Internal Medicine, Faculty of Medicine, Kindai University, Osaka-Sayama, Japan; d Department of Cardiovascular Medicine, Nippon Medical School, Tokyo, Japan

**Keywords:** Asia, ICD, primary prevention, sudden death, underuse

## Abstract

The effectiveness of primary prevention implantable cardioverter-defibrillators (ICDs) is well established. However, there are several unsolved problems related to ICD use for primary prevention in Asia, including ICD underuse, population differences in underlying heart disease, and the rate of appropriate ICD therapy compared with Western countries. Although the prevalence of ischemic cardiomyopathy in Asia is lower than in Europe and the United States, the mortality rate of Asian patients with ischemic heart disease has been increasing recently. As for the use of ICDs for primary prevention, there have been no randomized clinical trials, and limited data are available in Asia. This review focuses on the unmet needs related to ICD use for primary prevention in Asia.

## Highlights

• ICD is the standard care for primary prevention in patients with structural heart disease and high risk of SCD.
• There are unmet needs in Asia, including ICD underutilization, the lower prevalence of ICM and SCD compared to Western countries.
• This review also focused on the lack of data about the risk stratification of non-ICM, HCM, and CS in Asia.
• Large scale cohort studies or RCTs tackled with these issues in Asian patients are required in the future.

## Underuse of Prophylactic Implantable Cardioverter-Defibrillators in Asia

**Figure 1:**
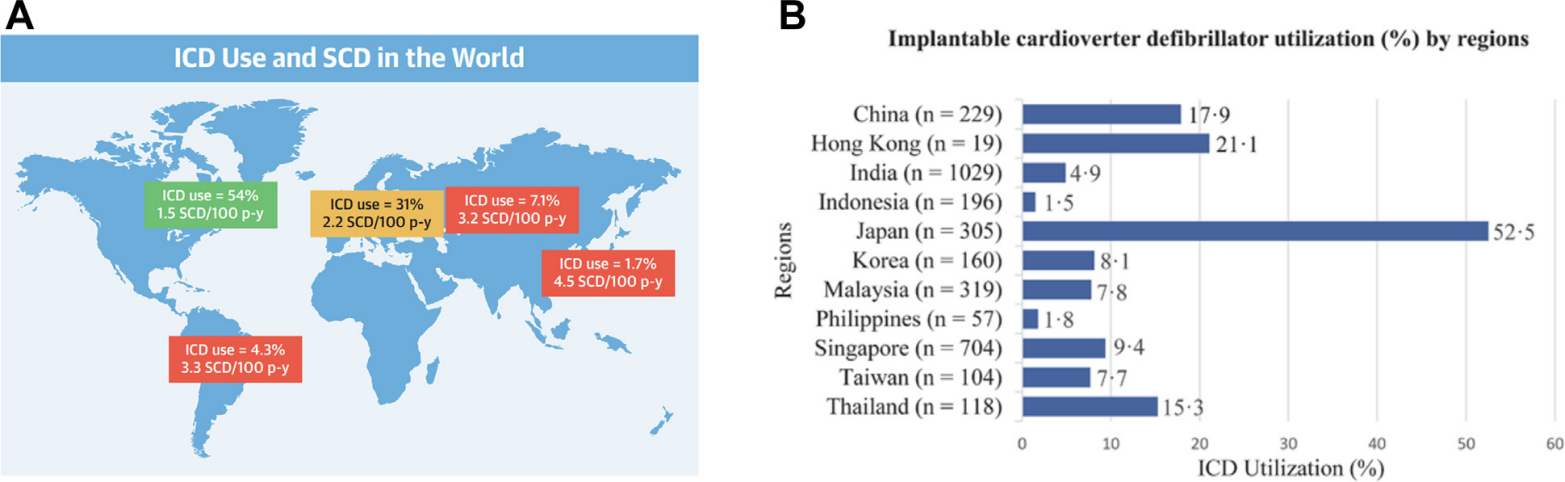
ICD Use in Asia and Western Countries **(A)** Implantable cardioverter-defibrillator (ICD) use and sudden cardiac death (SCD) rates worldwide from the PARADIGM-HF (Prospective Comparison of ARNI With an ACE-Inhibitor to Determine Impact on Global Mortality and Morbidity in Heart Failure) trial.^2^ ICD use varied by region, with the highest rates in North America (56%) and the lowest in Asia-Pacific (1.7%). **(B)** ICD use (percentage) by region. ICD use varied across Asia, with the highest in Japan (52.5%) and the lowest in Indonesia (1.5%).^3^

Although implantable cardioverter-defibrillator (ICD) insertion is an established therapy for primary prevention patients with structural heart disease, the underuse of ICDs in Asia is an issue that remains to be addressed. Recently, Rohde et al^1^ reported that there was marked geographic variation in the prevalence of ICD insertion on the basis of a subanalysis of the PARADIGM-HF (Prospective Comparison of ARNI With an ACE-Inhibitor to Determine Impact on Global Mortality and Morbidity in Heart Failure) trial.^2^ This study revealed that North America was the region with the highest ICD insertion rate (54%), and Asia-Pacific was the region with the lowest rates of ICD use (1.7%)^1^ (Figure 1A). Chia et al^3^ also showed that ICD use for primary prevention varied across Asia (from 1.5% in Indonesia to 52.5% in Japan) on the basis of the ASIAN-HF (Asian Sudden Cardiac Death in Heart Failure) registry (Figure 1B). In that report, the prevalence of ICD was 52.5% in Japan, 17.9% in China, and 8.1% in Korea. Among 5,625 KorAHF (Korean Acute Heart Failure) registry patients from 10 tertiary hospitals, only 56 patients (11.5%) underwent ICD insertion among 485 patients potentially indicated for ICDs for primary prevention.^4^ These data demonstrate that ICD use in Asia was lower than in Europe (24%)^5^ and the United States (30%-50%).^6^, ^7^, ^8^

It has been reported that higher ICD use is directly related to higher socioeconomic and national economic status and that lack of knowledge about device therapy was a barrier to patient acceptance of ICD insertion.^3^ The most common refusal reason was inability to pay for the device (53.8%), followed by not believing in the benefits of the ICD (19.4%) in the Improve SCA (Improve Sudden Cardiac Arrest) study, which included Asia.^9^ Hence, there were 3 barriers to ICD for primary prevention, including government level, health care provider level, and patient level. At the government level, rapid socioeconomic and epidemiologic transitions have occurred in Asia. Patients in low- and middle-income countries receive minimal to no reimbursement for ICD therapy, and reimbursement is restricted to secondary prevention implantations.^10^ In addition, out-of-pocket expenses tend to be higher in low-income regions such as Indonesia (46.9%), the Philippines (53.7%), and India (62.4%),^11^^,^^12^ where ICD use was low, contributing to the disparity in ICD use in Asia. At the health care provider level, physician unawareness^13^^,^^14^ was the contributing factor to underuse in Asia. Previous studies have revealed that physician awareness of the indications for ICD therapy is low,^13^^,^^14^ which leads to low referral rates and underuse of ICD insertion.^5^^,^^14^^,^^15^ As Asian patients tend to rely on their physicians for health information and are more accepting of ICD therapy if their physicians strongly recommend it,^16^ physicians should recommend ICD insertion for patients with heart failure (HF) with optimal ICD eligibility. At the patient level, a lack of knowledge about the preventive role of ICD was a main reply among patient responses to the questionnaire. As one-third of ICD nonrecipients (32.6%) were uncertain if devices could improve their quality of life and survival in a previous study, and knowledge of the role of ICDs for primary prevention was the most important factor influencing patient decisions, better patient education and physician recommendation could improve access.^16^ Physicians and government officials in Asia should address these fundamental issues.

## ICDs for Primary Prevention

The use of ICDs is effective in reducing life-threatening ventricular arrhythmia and preventing sudden cardiac death (SCD) for patients with structural heart disease, and their efficacy has been demonstrated not only for secondary prevention^17^, ^18^, ^19^ but also for primary prevention patients. In MADIT-II (Multicenter Automatic Defibrillator Implantation Trial), 1,232 patients with prior myocardial infarctions and left ventricular ejection fractions (LVEFs) < 30% were assigned in a 3:2 ratio to receive ICDs or conventional medical therapy, which removed nonsustained ventricular tachycardia (NSVT) and induction with electrophysiological methods as inclusion criteria. The study demonstrated that prophylactic ICD therapy reduced death of any cause (31%) during a mean observation period of 20 months.^20^ The SCD-HeFT (Sudden Cardiac Death in Heart Failure Trial) assigned 2,521 patients with NYHA functional class II or III congestive HF and LVEF ≤35% to conventional therapy plus placebo, conventional therapy plus amiodarone, or conventional therapy plus a conservatively programmed, shock-only, single-lead ICD, which excluded NSVT as an inclusion criterion, and ICD therapy was associated with a 23% decreased risk for death compared with the placebo and amiodarone groups after 5 years.^21^ These studies support the effectiveness of prophylactic ICD insertion for patients with coronary artery disease (CAD) and left ventricular (LV) dysfunction.

Although the data supporting the use of ICDs are robust in patients with ischemic cardiomyopathy (ICM), the benefits in patients with nonischemic cardiomyopathy (NICM) vary among clinical studies and meta-analyses. The DEFINITE (Defibrillators in Non-Ischemic Cardiomyopathy Treatment Evaluation) trial included 458 patients with nonischemic dilated cardiomyopathy, LVEF <36%, and premature ventricular complexes or NSVT, and ICD insertion was associated with a nonsignificant reduction in death of any cause (35%; *P* = 0.08).^22^ SCD-HeFT showed that ICD therapy reduced the risk for death among all cohorts, but the total mortality of NICM was less than that of ICM (NICM, 27% at 5 years; ICM, 43% at 5 years). Moreover, although the ICD group had 27% reduced total mortality compared with the placebo group, the difference was not significant (*P* = 0.06). The DANISH (Danish Study to Assess the Efficacy of ICDs in Patients with Non-Ischemic Systolic Heart Failure on Mortality) included 556 patients with LVEF ≤35% not caused by CAD that were randomly assigned to ICD vs non-ICD and revealed that the overall mortality rate was reduced by 13% but was not significantly different between the 2 groups (*P* = 0.28) during a median follow-up period of 68 months.^23^ Meanwhile, a meta-analysis of the effects of ICD therapy for NICM, including 5 randomized clinical trials enrolling 2,146 patients (DEFINITE, SCD-HeFT, CAT [Cardiomyopathy Trial],^24^ AMIOVIRT [Amiodarone Versus Implantable Defibrillator],^25^ and COMPANION [Comparison of Medical Therapy, Pacing, and Defibrillation in Heart Failure]^26^), showed that ICD insertion significantly reduced relative mortality (31%; *P* = 0.002).^27^ This result was also demonstrated in a recent meta-analysis of 6 trials enrolling 2,970 patients with NICM, with the addition of DANISH, indicating that ICD insertion significantly reduced relative mortality (23%) for patients with NICM.^28^ In addition, a meta-analysis of 5 randomized clinical trials including 2,573 patients with the addition of DANISH instead of COMPANION also found that relative mortality was significantly reduced by ICD therapy (21%; *P* < 0.001).^29^ These results may suggest that ICD therapy is effective for preventing sudden death in patients with NICM with LV dysfunction and with HF symptoms, as observed in ICM. However, large-scale data collection, including randomized clinical trials, has been conducted in only Europe and the United States, and at present, there are limited data supporting the use of ICDs for primary prevention in Asia.

## Primary Prevention vs Secondary Prevention in Asia

The risk for appropriate ICD therapy and prevalence of primary prevention varied among Asia, Europe, and the United States.^30^ Primary prevention patients had a lower risk for appropriate ICD therapy than secondary prevention patients in Europe, which was the same among patients in Asia, but the prevalence of primary prevention patients compared with secondary prevention patients is lower in Asian than in Western countries. In the Nippon Storm Study, which included ischemic and nonischemic patients with LVEF <50% in Japan,^31^ primary prevention patients (n = 531) showed a significantly lower risk for cumulative incidence of appropriate therapy than secondary prevention patients (n = 454) (*P* = 0.001), in concordance with a previous study. In Korea, the appropriate ICD therapy rate, including shock and antitachycardia pacing, was significantly higher in the secondary prevention group.^32^ Thus, the rate of appropriate ICD therapy was lower in primary prevention patients than in secondary prevention patients, consistent with data from Western countries. Among primary prevention patients, appropriate therapy rates were reported to range from 9% over 30 months to 37% over 60 months^30^^,^^33^ in Western countries, which was comparable with those for Asian patients, which ranged from 10% over 24 months to 39% over 42 months^31^^,^^32^^,^^34^^,^^35^ (Table 1).

**Table 1:** ICD Therapy for Primary and Secondary Prevention in Western Countries and Asia

	Sabbag et al.^33^	van Welsenes et al^30^	Aonuma et al.^37^	Kabutoya et al.^51^	Kotake et al.^31^	Yokoshiki et al.^36^	Cho et al.^4^	Park et al.^32^
n	2,349	2,134	171	392	985	17,564	305	146
Year	2015	2011	2022	2021	2021	2020	2020	2017
Country	Israel	the Netherlands	Japan	Japan	Japan	Japan	Korea	Korea
Primary/secondary, %	75/25	61/39	100/0	42/58	54/46	26/74	55/45	25/75
ICM/NICM, %	84/16	70/30	42/58	100/0	42/58	36/64	43/57	50/50
Appropriate ICD therapy/ICD shock	3.9/1.1 (1 y)	37 (5 y)/20 (5 y)	10 (24 mo)/NA	20 (20 mo)/9 (20 mo)	22 (36 mo)/NA	NA	18/12 (31 mo)	NA/39 (42 mo)

ICD = implantable cardioverter-defibrillator; ICM = ischemic cardiomyopathy; NA = not available; NICM = nonischemic cardiomyopathy.

The prophylactic use of ICDs is reported to be low in Asia, as mentioned earlier. Patients in Japan and Korea had a relatively high prevalence of primary prevention ICD, ranging from 25.6% to 64%,^30^^,^^33^^,^^36^ whereas the rate ranges from 61% to 75% in Western countries.^34^^,^^37^ The proportion of primary prevention patients was only 44.4%, even among patients with Class 1 indications for ICD, and 8.7% for those with Class 2 indications, according to the CHART-2 study of a chronic HF cohort in Japan.^38^ Thus, underuse of prophylactic ICD insertion in Asia, including Japan and Korea, has been an issue. Primary prevention is not sufficiently widespread in Asia, and it is thus important to encourage a strategy of prophylactic ICD insertion for patients with structural heart disease and ICD indications.

## SCD in Eastern Countries vs Western Countries

**Figure 2:**
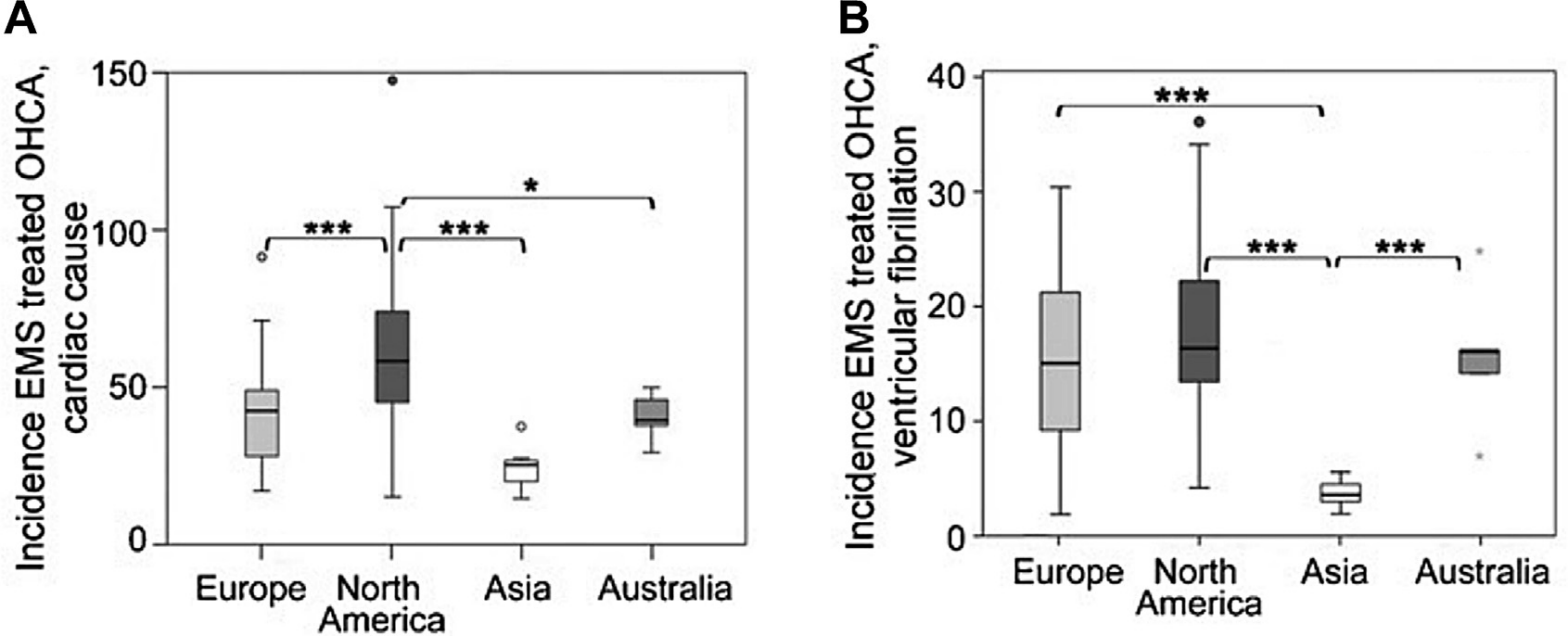
Incidence of EMS-Treated OHCA Incidence and survival rate per emergency medical services (EMS)–treated out-of-hospital cardiac arrest (OHCA) of presumed cardiac etiology **(A)** and with ventricular fibrillation **(B)**.^40^ The incidence of EMS-treated OHCA of presumed cardiac etiology was higher in North America (54.6) than in Europe (35.0), Asia (28.3), and Australia (44.0) (*P* < 0.001). The incidence of EMS-treated OHCA with ventricular fibrillation was lower in Asia (3.2) than in Europe (12.8), North America (14.0), and Australia (14.9) (*P* < 0.001). ∗*P* < 0.05 and ∗∗∗*P* < 0.001.

SCD is an unexpected death of cardiac causes occurring within a short time period (generally within 1 hour of the onset of symptoms if witnessed or within 24 hours of having been observed alive if unwitnessed) in a person without any prior condition that would appear fatal.^39^ In previous reports, the prevalence of SCD was low in Asia. From a systematic review of 67 studies, the incidence of out-of-hospital cardiac arrest of presumed cardiac etiology per 100,000 people per year was estimated to be 35.0 in Europe, 54.6 in North America, 28.3 in Asia, and 44.0 in Australia^40^ (Figure 2). In Asia, the incidence of SCD per 100,000 people per year was reported as 37 in Japan,^41^ 41 in China,^42^ 38 in Thailand,^43^ and 43 in the Philippines,^44^ all of which are relatively lower than in the United States and Europe. U.S. vital statistics mortality data from 1989 to 1998 showed that Asians had a lower incidence of SCD than African Americans and Whites; namely, SCD accounted for 63.7% of all cardiac deaths among Whites, 62.0% among Blacks, 60.5% among American Indians/Alaska Natives; and 55.2% among Asian/Pacific Islanders.^45^

Most SCD is caused by myocardial infarction and subsequent ventricular fibrillation in out-of-hospital cardiac arrest, but the proportion of ICM among patients with SCD in Asia is lower than in Western countries.^46^ The Hisayama study, a prospective population-based study of cardiovascular disease that has been under way since 1961 in Japan, revealed that ischemic heart disease was the most common cause of SCD according to autopsy data, but the rate was 28.9% among sudden death victims.^47^ In Hong Kong, 289 SCD victims were evaluated, and the major cause of death was CAD (35%).^48^ The rates of CAD among SCD victims were lower than in the United States (62%).^45^ The POST SCD (Postmortem Systemic Investigation of SCD) study revealed that the incidence of SCD was intermediate in Asians and Whites, whereas the proportion of sudden arrhythmic death was highest in Asians (61.9%) and lowest in Blacks (44.6%).^49^ This evidence indicates that the causes of SCD in Asia are somehow different from those in Western countries; that is to say, the causes of SCD are heterogeneous and include ICM and NICM, hypertrophic cardiomyopathy (HCM), cardiac sarcoidosis (CS), and primary electric heart disease.^50^

## Ischemic Cardiomyopathy

Previous studies showed that both the prevalence of patients with ICM and the incidence of SCD were low in Asians. The prevalence of patients with ICM among primary prevention ICD was lower in Asia (36%-50%)^4^^,^^31^^,^^32^^,^^36^^,^^51^ than in Western countries (70%-75%),^2^^,^^52^ which was 50% at most in Asia but more than 60% in Western countries. This could be due to the difference in prevalence of underlying heart disease. The CHART-1 study, which enrolled 1,278 consecutive patients with congestive HF in stable condition between 2000 and 2005, revealed that the most prevalent etiology of HF was NICM (28.6%), and CAD accounted for 25.4% of total patients with HF.^53^ This ischemic HF prevalence was considerably low compared with that reported in Western studies.^54^ In the primary percutaneous coronary intervention era, there remains a low incidence of SCD in Asians,^55^ but the prophylactic use of ICD remains effective for primary prevention patients with LV dysfunction. The JID-CAD (Japan Implantable Devices in Coronary Artery Disease) study, which included CAD and ICD or cardiac resynchronization therapy device patients in Japan, revealed that the rates of appropriate ICD therapy in the primary (n = 165) and secondary (n = 227) groups were similar during the follow-up period.^51^ The presence of appropriate ICD therapy was 37% at 3 years in the Japanese cohort using MADIT-II criteria for patients with ICM with primary prevention ICDs.^56^ A subanalysis of the Nippon Storm Study demonstrated that the incidence of ICD therapy in patients with CAD for primary and secondary prophylaxis was not significantly different.^57^ Even though the incidence of SCD due to CAD is low in Asians, the efficacy of prophylactic ICD is comparable with secondary prevention ICD.

**Figure 3:**
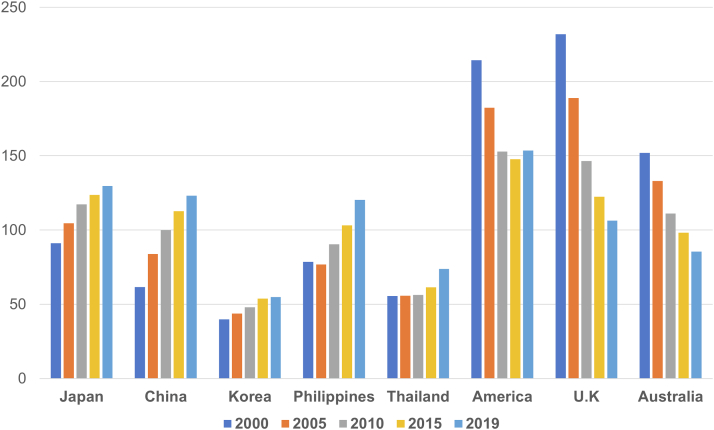
The Incidence of Death of Ischemic Heart Disease The incidence of death of ischemic heart disease per 100,000 people per year on the basis of World Health Organization data on the leading causes of death. The incidence of death of ischemic heart disease was more than 2- to 3-fold in Western countries compared with Asian countries in 2000, but recent data showed that the gaps are closing between Western and Asian countries.

In contrast, the Hisayama study showed that the prevalence of ischemic heart disease among the causes of SCD increased significantly with time.^47^ Data from the World Health Organization regarding the leading causes of death showed that the incidence of death of ischemic heart disease per 100,000 people per year was 91.1 in Japan, 61.5 in China, 39.7 in Korea, 231.8 in the United Kingdom, and 214.4 in the United States, more than 2- to 3-fold in Western countries compared with Asian countries in 2000. However, recent data has showed that these gaps are closing between Western countries and Asian countries, with 129.6 in Japan, 123.02 in China, 54.8 in Korea, 106.3 in the United Kingdom, and 153.5 in the United States^58^ (Figure 3). The CHART-2 study, which enrolled 10,219 consecutive patients with symptomatic and asymptomatic HF between 2006 and 2010, revealed that the prevalence of CAD in patients with stage C/D HF (n = 4,735) increased to 47.1% from 25.4%, demonstrating the rapid westernization trend in the etiology and clinical characteristics of patients with HF in Japan,^59^ which is comparable with China.^60^ Thus, rigorous consideration of primary prevention ICD indications for patients with ICM with LV dysfunction will become much more important in the future, as SCD is expected to occur more frequently as the rate of ICM increases.

## Nonischemic Cardiomyopathy

The DANISH study questioned the efficacy of prophylactic ICD, as it showed that ICD insertion did not significantly decrease the overall mortality rate in patients with NICM.^23^ However, 3 meta-analyses demonstrated that ICD insertion significantly reduced the relative mortality.^27^, ^28^, ^29^ In addition, combined data from 5 landmark ICD clinical trials (MADIT-II, MADIT-CRT, MADIT-RIT, MADIT-II RISK, and RAID) showed that patients with ICM and those with NICM experienced a similar risk for ventricular tachycardia or ventricular fibrillation events.^61^ In Italy (n = 1,964)^62^ and France (n = 5,539),^63^ patients with ICM and those with NICM had a similar risk for ventricular arrhythmia in a large cohort of patients with primary prevention ICDs for SCD. Meanwhile, in Asia, the ICD cohort from the entire Korean population in the Korean National Health Insurance Service database (n = 1,097) revealed that the rate of arrhythmic death did not differ significantly between the ICM and NICM groups.^64^ Although several reports showed the benefit of prophylactic ICD patients with NICM, the results of the DANISH study did not reach statistical significance. It is speculated that this may be due to the older age of the cohort. Younger patients may be more prone to ventricular tachyarrhythmia, whereas older patients may be more likely to die of pump failure or noncardiovascular reasons.^65^ In a subanalysis of the DANISH study, ICD insertion was associated with reduced all-cause mortality in patients ≤70 years of age.^66^

An epidemiologic transition has occurred in Asia along with aging of the population, and the susceptible age for patients with HF was heterogeneous in each region.^54^ Registered patients were relatively younger in China (median age 60-62 years) and Southeast Asia (median age 53, 60, 61, 67, and 71 years for the Philippines, Indonesia, Malaysia, Thailand, and Singapore, respectively) compared with Japan, Taiwan, and Korea (median age 67-73, 73, and 69 years, respectively).^67^ Thus, the age spectrum of SCD and HF is somehow different between Western and Eastern countries, and further investigation is needed to determine the optimal cutoff age for the benefit of prophylactic ICD in Asia.

## Hypertrophic Cardiomyopathy

Primary prevention ICD for HCM contributes to lower SCD rates than the previous era.^68^ Contemporary HCM-related SCD rates have been low (0.32% per year) from 2015 to the present compared with 2000 or earlier. As for the global trend, reported SCD rates for HCM were lowest in North America (0.15% per year) and highest in Asia (0.61% per year), although the incidence of SCD decreased over time in each region.^69^ In addition, in a study of a total of 1,661 consecutive patients (1,210 European patients and 451 Asian patients, including 308 Chinese, 83 Malay, and 60 South Asian patients) diagnosed with HCM, the number of ICDs was significantly lower in the Asian patients (2% vs 19%). The event rates for the overall survival and the combined endpoint survival and ICD shocks were significantly higher in the Asian patients. Thus, SCD rates are assumed to be high in Asia.

It is unknown whether there is a racial difference in SCD risk in patients with HCM, which is important in terms of risk stratification. There was no significant difference in SCD in U.S. vs non-U.S. patients (Italy, the Netherlands, the United Kingdom, Brazil, and Australia) who did not receive ICDs, though there was a lower rate of appropriate ICD therapy at U.S. sites.^70^ The data showing that primary prevention ICD use was higher and that there were higher rates of lower risk populations in the United States than elsewhere were associated with a lower rate of appropriate ICD therapy. In addition, there is no significant difference in the rate of ICD-appropriate therapy among Asia (6.6%),^71^ the United States (6.7%), and non-U.S. countries (8.4%). These results suggest that the risk for SCD is equivalent for each race and that prophylactic ICD could be equally valid.

There are various conflicting guidelines and risk stratifications of SCD for HCM. Two major SCD risk stratification methods have been recommended by American and European experts or guidelines for the management of HCM. The 2011 and 2017 American College of Cardiology (ACC) Foundation/American Heart Association (AHA) guidelines recommended stratifying patients on the basis of a simple summation of several established SCD risk markers (family history of SCD, LV wall thickness ≥30 mm, unexplained syncope for <6 months, NSVT, and abnormal blood pressure response during exercise) and SCD risk modifiers (age <30 years, late gadolinium enhancement on cardiac magnetic resonance imaging, and LV outflow tract obstruction).^72^^,^^73^ The 2014 European Society of Cardiology guidelines recommended the stratification of patients by calculating the 5-year SCD risk score according to a mathematical model using maximum LV wall thickness, left atrial diameter, maximum LV outflow tract gradient, family history of SCD, unexplained syncope, and age (HCM Risk–SCD model).^74^ The utility of the 2011 ACC/AHA guideline and HCM Risk–SCD model has been reported in China,^75^ Japan,^76^ and Korea.^77^ In Japan, both the summation of SCD risks and the HCM Risk–SCD model are used for the recommendation of prophylactic ICD therapy for HCM.^78^ Prophylactic ICD indications for HCM should be considered prudently, taking this background into account.

## Cardiac Sarcoidosis

There has been a marked increase over time in the number of new CS diagnoses in the MIDFIN (Myocardial Inflammatory Diseases in Finland) registry.^79^ Many patients presented with several parallel manifestations of cardiac disease, such as atrioventricular block, SCD, and LV dysfunction.^80^ SCD was the main cause of death, constituting up to 80% of all deaths, but the indication for prophylactic ICD differs between the ACC/AHA guidelines^73^ and the Japanese Circulation Society guideline.^81^ In the ACC/AHA guideline, ICD insertion is a Class 2a recommendation for patients with CS who have indications for permanent pacing. In contrast, in the Japanese Circulation Society guidelines, patients with an LVEF of <50% who are indicated for pacemaker implantation and who either have NSVT or in whom sustained ventricular tachycardia or ventricular fibrillation was induced during electrophysiological testing have a Class 2a recommendation for ICD. One study compared outcomes in patients with high-degree atrioventricular block as the initial manifestation of CS with those in patients who initially presented with ventricular tachycardia and/or HF, showing that fatal cardiac events, including sustained ventricular arrhythmias, were similar to those with ventricular tachycardia and/or HF, suggesting that the risk for fatal cardiac events is high, regardless of the initial clinical presentation and steroid use.^82^^,^^83^ Another retrospective study of CS in Japan revealed that patients with Class 2a indication for ICD and inducible sustained ventricular arrhythmia had no SCD and ventricular arrhythmia events, which may indicate that electrophysiology is not useful for SCD risk stratification of CS.^84^ Although the ACC/AHA guidelines (without the statement for NSVT and electrophysiology) is valid for Asian patients with CS, further study is needed for SCD risk stratification.

## Conclusions

This review focused on the issue of prophylactic ICD for structural heart disease in Asia and clarified ICD underuse, the lower prevalence of SCD, and the lower rate of ICM compared with Western countries. As there have been limited data on the risk stratification of NICM, HCM, and CS in Asia, further investigation is needed, especially to determine the optimal cutoff age of ICD indication in NICM, a risk stratification algorithm for HCM, and the necessity of NSVT and electrophysiology for ICD indication in CS (Central Illustration). In addition, large-scale randomized clinical trials have been conducted only in Western countries, and trials in Asian patients with structural heart disease are thus needed to achieve a better understanding.

**Central Illustration:**
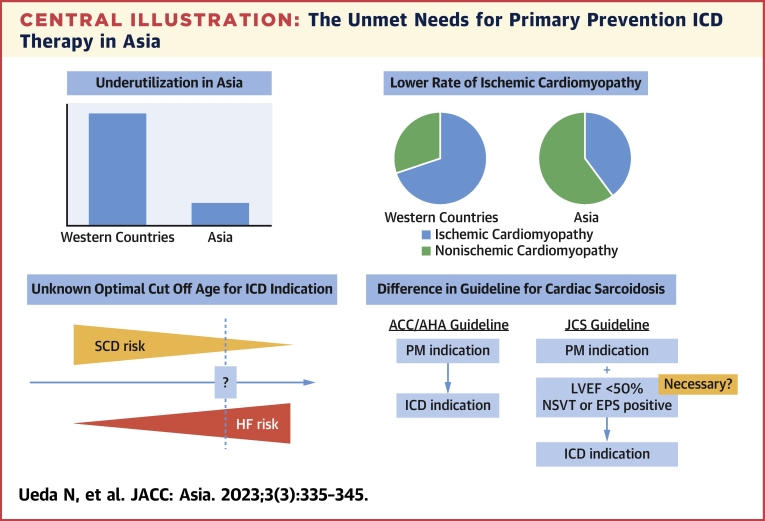
The Unmet Needs for Primary Prevention ICD Therapy in Asia This illustration shows the unmet needs for primary prevention implantable cardioverter-defibrillator (ICD) therapy in Asia, namely, ICD underuse, a lower rate of ischemic cardiomyopathy, limited data on the optimal cutoff age for ICD indication in nonischemic cardiomyopathy, and the necessity of nonsustained ventricular tachycardia (NSVT) and electrophysiological study (EPS) for an ICD indication for cardiac sarcoidosis. ACC = American College of Cardiology; AHA = American Heart Association; HF = heart failure; JCS = Japanese Circulation Society; LVEF = left ventricular ejection fraction; PM = pacemaker; SCD = sudden cardiac death.

## Funding Support and Author Disclosures

This study was supported by the intramural research fund (25-4-7, Kengo Kusano) for cardiovascular diseases of the National Cerebral and Cardiovascular Center. Dr Noda was partially supported by Japan Society for the Promotion of Science KAKENHI grants 19K08570 and 22K08092 to conduct this study. This study was supported by the National Center Consortium in Implementation Science for Health Equity, funded by the Japan Health Research Promotion Bureau Research Fund (2019-[1]-4). Dr Ueda has received honoraria for lectures from Medtronic Japan. Dr Noda has received honoraria from Medtronic Japan and Biotronik Japan; and is a member of a department endowed by Biotronik Japan. Dr Kusano has received honoraria from Biotronik Japan and Medtronic Japan; and has received research grants from Medtronic Japan. Dr Yasuda is a member of a department endowed by Abbott Japan, Nihon Kohden, Japan Lifeline, Medtronic Japan, and Biotronik Japan. Dr Kurita has received honoraria from Medtronic Japan, Abbott Japan, Japan Lifeline, and Biotronik Japan. Dr Shimizu has received honoraria from Medtronic Japan.
